# A Program Evaluation of a Dietary Sodium Reduction Research Consortium of Five Low- and Middle-Income Countries in Latin America

**DOI:** 10.3390/nu14204311

**Published:** 2022-10-15

**Authors:** Janice Padilla-Moseley, Adriana Blanco-Metzler, Mary R. L’Abbé, JoAnne Arcand

**Affiliations:** 1 Faculty of Health Sciences, Ontario Tech University, 2000 Simcoe Street North, Oshawa, ON L1G 0C5, Canada; 2 Costa Rican Institute of Research and Teaching in Nutrition and Health (INCIENSA), Tres Ríos P.O. Box 4-2250, Costa Rica; 3 Department of Nutritional Sciences, University of Toronto, 1 King’s College Circle, Toronto, ON M5S 1A8, Canada

**Keywords:** program evaluation, diet, nutrition, sodium, policy, public health, knowledge translation, research consortium, community of practice

## Abstract

Excess dietary sodium is a global public health priority, particularly in low- and middle-income countries where rates of hypertension and cardiovascular disease are high. The International Development Research Centre funded a research consortium of five Latin American countries (LAC) to inform public health policy for dietary sodium reduction (2016–2020). The objective of this study was to determine the outcomes of this funding on short-term (e.g., research, capacity building) and intermediary outcomes (e.g., policies). A summative program evaluation was conducted, using a logic model and multiple data sources including document review, surveys and interviews. Researchers from Argentina, Costa Rica, Brazil, Peru and Paraguay produced a significant amount of scientific evidence to guide decision making on sodium policy related to its content in foods, consumer behaviors (social marketing), and the health and economic benefits of dietary reduction. A substantive number of knowledge translation products were produced. The funding enabled training opportunities for researchers who developed skills that can be scaled-up to other critical nutrients and health issues. It was unexpected that intermediary policy changes would occur, however several countries demonstrated early policy improvements derived from this research. A funded research consortium of LAC is a practical approach to invoke policy innovations.

## 1. Introduction

Low-and middle-income countries (LMIC) are disproportionately impacted by non-communicable diseases (NCD). Globally, two thirds of deaths annually are attributed to NCDs; while LMICs experience four-fifths of these deaths predominantly due to cardiovascular diseases (CVDs) [1]. NCDs have a high economic burden in LMICs, where direct costs are significantly higher compared to higher income countries [2]. Additionally, several NCD risk factors, including hypertension, are more common in LMICs [3] which adds substantive social and economic burden from NCDs in these resource-constrained settings [4].

Excess dietary sodium is a leading risk factor for NCDs, including hypertension, CVDs, stroke and kidney disease [5,6]. Globally, excess sodium intake is associated with approximately 3.2 million deaths annually and 70 million disability-adjusted life-years [7,8]. Reducing dietary sodium lowers blood pressure and risk of hypertension, CVD and stroke-related deaths, as well as other NCDs related with excessive sodium intake [5,6,9]. The World Health Organization (WHO, Geneva, Switzerland) recommends adults consume less than 2000 mg of sodium/day (equivalent to 5 g of salt/day) [10]; however, intakes in LAC far exceed these recommendations. For example, the average estimated sodium intake in Argentina is 4480 mg/day [11,12,13], Brazil is 4720 mg/day [14], Costa Rica is 4600 mg [15], Paraguay is 5480 mg/day [16] and Peru is 3880 mg/day [17]. Dietary sodium sources vary by country. In most LAC, a large proportion of dietary sodium is derived from discretionary sodium (i.e., sodium added via salt during cooking or at the table) [15,18]; whereas in some countries the primary source is from packaged and prepared foods [12].

The WHO set global targets to address NCD risk factors, which included a 30% relative reduction in mean population sodium intake by 2025 [3]. Policy interventions to support dietary sodium reduction are highly cost effective and thus have been labelled as “Best Buys” by the WHO [19]. Not surprisingly, the number of countries with a national level sodium reduction program have increased by 28% since 2014 [20]. These national sodium reduction programs are most often multi-component, incorporating a combination of the WHO’s Best Buy interventions, including food reformulation with target sodium levels, consumer education, healthy foods in public settings, and front-of-pack labelling [20]. However, despite country-level commitments to sodium reduction, progress towards the global targets is lagging. Only a few countries have reduced population sodium intakes and none have met WHO’s global target for sodium reduction [20,21].

In Latin American countries (LAC), policy development and implementation are complex and often challenging. Up-to-date national-level data that is high quality is frequently unavailable. This includes key data to drive sodium policy such as on the health and economic benefits of sodium reduction policies, the sodium content of foods consumed nationally and the factors influencing dietary sodium behaviors. A recent qualitative study demonstrated that the limited availability of sodium research in LAC is often due to a lack of funding, and limited human resources and infrastructure available to researchers [22]. Researchers in LAC are also overstretched with their time, which limits their ability to effectively translate research findings to policy decision makers [22]; a commonly documented barrier related to nutrition policy research in LMICs in general [23,24].

In 2015, the International Development Research Centre (IDRC), a Crown corporation of the Canadian federal government, created a “Food, Environment and Health” research funding program. The funds were in support of research on healthy food systems in LMICs, with an aim of reducing the health, social and economic burdens of diet-related NCDs. Under this program the IDRC funded a five country Latin American consortium to conduct research to inform public health policy innovations for dietary sodium reduction (IDRC Grant 108167). The consortium was led by the Costa Rican Institute of Research and Teaching in Nutrition and Health (INCIENSA, Principal Investigator: A.B.M) with support from the University of Toronto and Ontario Tech University. The consortium included numerous researchers from Argentina, Brazil, Paraguay and Peru and stakeholders from non-governmental organizations across the region [25]. Multiple research program objectives were funded, focusing on assessing the sodium content of packaged foods using food labels (program objective 1A); assessing the sodium content levels in artisanal, street and fast foods using chemical analysis (program objective 1B); creating a social marketing strategy based on research conducted to identify local barriers and facilitators to individual-level sodium reduction (program objective 2); assessing the health and economic impacts of population-wide sodium reduction (program objective 3); developing and executing country-specific knowledge translation strategies (program objective 4); and conducting a program evaluation of the funded consortium (program objective 5). Program objective 5 is the basis of this paper, which aims to share the overall impacts of the funded research program. Concurrently this paper offers a unique contribution to the literature by demonstrating if a funded research consortium in LMICs can effectively lead to advancements in dietary sodium reduction policies, among other benefits to the countries and the researchers [26]. The IDRC funding was administered from 2016 to 2020 to five LACs. It was hypothesized that this “intervention” would stimulate collaboration and capacity building in conducting research and accelerate dietary sodium reduction policy development and implementation, ultimately improving health outcomes. Specifically, this program evaluation assessed if the following consortium-level outcomes (i.e., short-term outcomes) were achieved:

1. The creation of scientific evidence and innovations across multiple research program objectives that can be scaled-up to produce policy changes across multiple LAC;
2. The formation of multi-sectoral and multi-disciplinary partnerships;
3. The formation of equitable, diverse and inclusive partnerships;
4. The enhancement of consortium researchers’ confidence, capacity, and scientific abilities in conducting research to address public health nutrition issues in LACs.

The program evaluation also assessed intermediate program outcomes which were defined as sodium reduction policy and program changes (e.g., a policy commitment from the food industry and addition of sodium reduction to a political agenda); however it was unexpected that would occur over the relatively short 3.5-year funding period.

## 2. Methods

**Figure 1:**
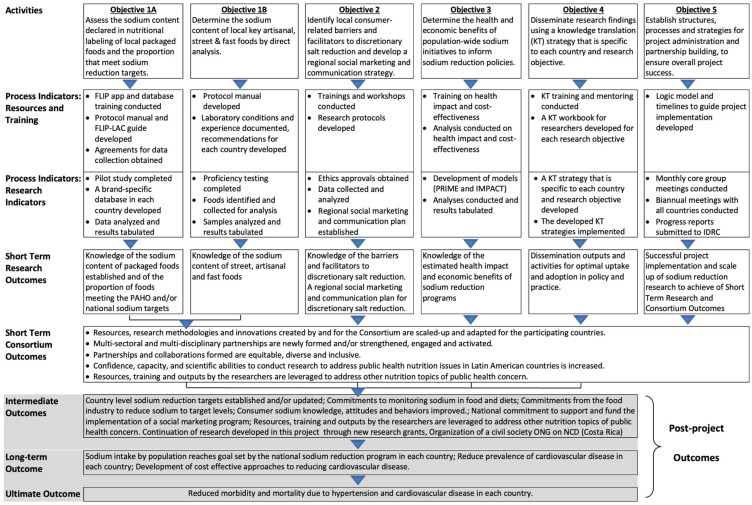
Research Program Logic Model.

A summative program evaluation took place at the end of the consortium funding period. The evaluation took a multiple method approach using quantitative and qualitative data from diverse sources consisting of a document review, program evaluation survey, team meetings and qualitative interviews. The program evaluation was guided by a logic model (Figure 1) developed in 2016, as well as the Public Health Agency of Canada’s Planning Public Health Programs [27] and the Centers for Disease Control’s program evaluation framework [28,29]. The logic model defined process indicators, consortium outcomes (i.e., short term outcomes), intermediate and long-term outcomes [30,31]. Consortium researchers provided iterative feedback of the logic model to ensure the LAC contextual factors were considered.

### 2.1. Data Collection

Program outcomes were evaluated with multiple methods. This data added a variety of insights to the evaluation, including social, cultural, and historical contexts on the research landscape in a country.

*Document Review*. Several types of documents were reviewed, including the original grant application, interim progress reports, a compendium of knowledge translation activities conducted, meeting notes, memos, and country-level data on existing programs and policies from the PAHO [32].

*Program Evaluation Survey*. In January 2020, a web-based survey was sent to the consortium researchers (*n* = 18). The survey evaluated consortium-level outcomes related to partnerships, capacity, and the overall experiences as a consortium researcher. The ESSENCE framework [33] and Larkan’s et al. (2016) [34] attributes informed the survey questions related to partnerships. Included were 10 multiple choice questions based on a 5-point Likert scale and eight open-ended questions. Two external independent reviewers assessed the survey for face and content validity. A response rate of 61% was achieved, with responses received each participating country.

*Team meetings*. In February 2019, the leadership team at INCIENSA hosted an interim team meeting with all consortium researchers and stakeholder partners. Researchers from each country presented preliminary study results and sought feedback and input from their peers and partners. During these meetings, challenges and successes with research were documented. In January 2020, a one-week face-to-face meeting in Costa Rica took place with the leadership team at INCIENSA, where they were interviewed about the project outcomes. Meeting notes were used to inform the evaluation report. Three additional informal interviews were conducted with consortium researchers, via video conference, to expand upon and add clarity to the outcomes assessed in this evaluation.

*Qualitative interviews*. From November 2019 to February 2020, a qualitative case study utilized one-on-one semi-structured interviews with the consortium leads (*n* = 5) and Ministry of Health officers (*n* = 4) from each participating country. The methodology and results have been described previously in another qualitative study [22]. The Ministry of Health officers were arm’s length to the research and were interviewed due to their role in policy decision making. The interviews were 45 to 60 min in duration and explored the barriers and facilitators to implementing sodium reduction policies and programs in the consortium countries. Themes from Trostle’s et al. (1999) [35] and the Diffusion of Innovation (DOI) Theory [36] informed the interview guide. Verbatim transcripts underwent a deductive thematic analysis where two independent researchers coded the transcripts.

### 2.2. Data Analysis

Qualitative data was analyzed using a thematic deductive analysis obtained from the documents, transcripts from meetings and interviews and open-ended survey responses. These data were organized and coded with Nvivo Software (Version 12), using codes and a codebook that were established a priori using the DOI, Trostle’s et al. (1999) themes and consortium and intermediate level outcomes from the logic model. Quantitative data were analyzed using descriptive statistics. Evaluation data were triangulated through multiple data sources that ensured validation, credibility and added to the depth and breadth of the findings. 

## 3. Results

The consortium consisted of researchers from the government, academia and NGO from Argentina, Brazil, Costa Rica, Paraguay and Peru. Each country had an assigned lead researcher (*n* = 5) who oversaw the research activities alongside other researchers and funded research assistants. The Costa Rican Institute for Research and Teaching in Nutrition and Health (INCIENSA) was the technical and financial coordination and leadership site and led the consortium (Principal Investigator A.B.M). A governance group provided scientific, knowledge translation, and project governance support to the consortium team and the principal researcher, which included representatives from the Pan American Health Organization (PAHO), the University of Toronto, and Ontario Tech University.

### 3.1. Consortium Outcomes (Short Term)

#### 3.1.1. Outcome 1: Research Conducted, Data Generated and Innovations Scaled-Up or Created

This evaluation outcome assessed the research conducted, data generated and innovations that were scaled-up in the consortium countries. Consortium countries chose which research objectives they participated in, which was impacted by national priorities and the availability (or lack of availability) of pre-existing research. Specifically, program objectives 1A to 4 were assessed:

*Program objectives 1A and 1B: Sodium Content of Packaged, Artisanal, Street and Fast Food Products* (Argentina, Brazil, Costa Rica, Paraguay, Peru). Comprehensive data on the sodium content of packaged foods was unavailable in some participating countries, and the extent to which they were meeting their national and/or the regional sodium reduction targets was unknown. In addition, a systematic and standardized approach with state-of-the-art technology for data collection and analysis of the sodium content of packaged foods was not available in LAC. These research gaps were addressed by adapting data collection and analysis innovations and implementing them across the consortium.

With the IDRC funding, the University of Toronto’s Food Label Information Program (FLIP) mobile application and web-based software for collecting data from packaged food labels was adapted for use by the consortium. The modified application/software was called FLIP-LAC and it included training manuals [37], guides [38] and standardized procedures on food label and data collection. The data generated from FLIP-LAC led to the development of large brand-specific databases containing the nutrient (sodium) composition of food products sold in each consortium country; except for Brazil which used a slightly different method that was considered more appropriate for their country context. The use of FLIP-LAC afforded a unique ability to collect not only information about sodium, but on all nutrients reported on food labels, the individual ingredients and on front-of-package labelling and marketing.

The use of FLIP-LAC led to the systematically collected and analyzed data on sodium content of 8314 packaged food products in four countries (with one package size per food product included in the analysis). Mean sodium content was assessed (mg/100 g) for commonly consumed sources of sodium, with the results being compared to 2015 PAHO regional targets. The latter analysis found that Paraguay had 87.9% of food products meeting regional sodium targets, followed by Argentina (87.0%), Peru (85.5%), Costa Rica (83.9%) [39]. In a separate analysis, it was found that 81.2% of Brazil’s food products met the regional sodium targets [40]. The findings also demonstrated high variability in the sodium content of foods within single food categories, suggesting high potential for reformulation of higher sodium products within a food category. This finding, along with the observed high proportion of products already meeting the regional sodium targets, highlighted the need for more stringent regional sodium reduction targets, to accelerate sodium intakes in LAC [39,40,41,42]. On completion of the IDRC grant, FLIP-LAC was scaled up and made broadly accessible across LAC through another funding source, and results were used to update PAHO regional targets.

Under program objective 1B, data on the sodium content of street, artisanal and fast foods were assessed using chemical analysis. The importance of this research is emphasized by the nearly complete lack of sodium content data on these culturally specific foods. Using standardized chemical analysis procedures and protocols, street and fast food products were sampled based on national consumption patterns and availability. A manual for food chemical analysis of sodium was created to standardize procedures and conditions for the analysis [43]. This was an important step given observation that there were highly variable experience levels of technicians and laboratory standards and procedures for chemical analysis across the consortium countries. The sodium content analysis on over 100 artisanal, street and fast food led to novel data on frequently consumed foods in LAC; demonstrating sodium levels in the moderate to high range. These data were submitted to the Latin American Network on Food Data System (LATINFOODS) database for researchers, nutritionists and food technologists to access.

Overall, program objectives 1A and 1B achieved a high degree of success with the evaluation outcomes, but some challenges were experienced. Challenges in obtaining approval for data collection from grocery stores led to setbacks and delays. This was a necessary step in all countries in order to initiate data collection. A high turnover rate of the grocery store staff contributed to communication break downs, where new staff had no knowledge transfer that authorization was received. Despite attempts at standardization and procedures to optimize analytic performance, some countries had variations in laboratory conditions and techniques for food chemical analysis which may impact the validity and comparability of the results. For example, some countries performed analysis with food additions such as salsas, seasonings, dressings and hot peppers; while other countries did not. Furthermore, the methodology used to estimate sodium varied in all countries.

*Program Objective 2: Social Marketing and Communication Strategy (Brazil, Costa Rica, Paraguay, Peru).* Since a relatively higher proportion of sodium intake comes from discretionary salt in LAC, it is essential to engage the public in changing their personal behaviors related to added sodium; however, little research had previously been conducted. Therefore, consortium researchers conducted formative research on consumer behaviors and created regional social marketing and communication strategies to drive population-wide behavioral changes related to dietary sodium. Expert social marketers and researchers from the University of South Florida led an innovative hybrid training program adapted for LAC with consortium researchers from Costa Rica, Brazil, Peru and Paraguay. The training consisted of a virtual course on social marketing concepts and qualitative data analysis, followed by face-to-face workshops. The training program was developed specifically for the LAC context and scaled-up to the consortium countries; where no other training program previously existed. After the training, consortium researchers prepared their own formative research proposals to conduct focus groups and interviews with a defined target audience to examine barriers, facilitators, beliefs, values and motivations related to discretionary salt use. These activities informed a regional social marketing and communication plan that was created by researchers at the University of South Florida. The social marketing training program and communication plan was a significant innovation created for the consortium countries. Multiple articles were published about this research [44,45]. After the consortium funding period, researchers at the University of South Florida developed a follow-up 4-module course for researchers interested in advanced-level social marketing training. All LAC can now access this training program on the PAHO Virtual Campus. Costa Rica is currently adapting the regional plan to their national context with funding from LINKS-Resolve to Save Lives.

*Program objective 3: Assessing the health and economic benefits of dietary sodium reduction.* Brazil and Costa Rica used health and economic data to quantify the attributable health impact and economic burden of excess sodium consumption in their countries. These data were not widely available in LAC, and few researchers had expertise to conduct such analyses. Two different modeling scenarios were applied. One model was the Preventable Risk Integrated ModEl (PRIME), developed by the University of Oxford, that was used to conduct analysis on avoidable deaths due to excess sodium. The other model used was IMPACT food model (economic impact), to conduct health and economic impact assessments on CVD deaths and cases prevented or postponed in different modeling scenarios related to sodium reduction over time [46].

A Brazilian researcher received in-person training on IMPACT at the University of Liverpool; training that was later relayed to the Costa Rican research team. Researchers from Costa Rica and Brazil both received face-to-face training on the use of PRIME from the Université Laval, followed by two webinars. The findings from this research were published [46,47,48,49], and used to form strong arguments with policy decision makers on setting upper limits on sodium in food products. In addition to the data generated, the capacity built can be applied towards the creation of health and economic data related to other public health priorities. These models provided a pragmatic and cost-effective way for countries with limited resources and missing data to conduct analysis. There were unexpected challenges with the use of the PRIME model as the consortium researchers required in-depth statistical analysis to review long term trends and risks over time. Furthermore, the IMPACT model required more data inputs which were not always available from health information custodians; requiring some assumptions to be made in the model.

*Program Objective 4: Knowledge translation (KT) strategies*. Most consortium researchers noted that formal KT strategies were a new concept and that culturally specific tools were needed. As part of the consortium, a customized KT workbook was developed to ensure maximal usability and uptake of research data. The KT workbooks considered the research and regional context, including translation of key terms in Spanish. The customized KT workbooks were peer reviewed, piloted and validated for use by the researchers, to optimize communication and knowledge dissemination tactics for their research. This was an important scalable innovation that can be used in future studies. Consortium researchers noted that KT training enabled their thinking about ways to engage policy makers earlier in the research process: “when we have any research grants for small funded projects, we have added something that was actually inspired by the IDRC project … is that this communication plan … this knowledge transfer. It is something that is very important for presenting the results, making the results easier to understand for any audience”. With the technical assistance of the InterAmerican Heart Foundation, a policy brief was developed in English and Spanish [50], which was disseminated to decision makers at Ministries of Health, the food industry and other key stakeholders. The benefits of policy briefs were also realized as part of the process. The Costa Rican researchers noted that they now embed policy briefs as a key dissemination output for their studies. A limitation to the KT training and KT plan development was that it was introduced early in the second year of the funding period, which is a relatively late stage to incorporate rigorous integrated KT strategies. KT strategy development at the onset of the grant would have involved policymakers and other stakeholders early in the research development process, to promote maximal impact of the data on policy development and implementation.

Most of the grey literature and papers generated from this consortium is published and available in IDRC Digital Library (https://idl-bnc-idrc.dspacedirect.org/discover (accessed on 15 August 2022)) which is available for policy makers and researchers.

#### 3.1.2. Outcome 2: Multi-Sectoral and Multi-Disciplinary Partnerships Were Newly Formed, Strengthened, Engaged and Activated

*Consortium researchers and governance committee.* The consortium included multi-sectoral and multi-disciplinary partnerships to drive dietary sodium research and policy. The PI, with support from PAHO, invited other several LMIC in Latin America to join the consortium, with Argentina, Brazil, Costa Rica, Paraguay and Peru expressing interest. During consortium formation, researchers from the University of Toronto, Ontario Tech University and PAHO provided technical expertise to the PI on the development of the grant application (e.g., writing support, protocol development). Under the leadership of the PI (ABM), an advisory governance committee was formed, including public health nutrition researchers, experts and from INCIENSA, the University of Toronto, Ontario Tech University and PAHO. This group established a framework to support the operations and decision making of the project. They held periodic meetings to support guidance on leadership, technical and scientific aspects of the consortium. Consortium researchers reported that PAHO, Canadian and European educational institutions were significant partners who supported research success.

*Partnerships formed to execute research and drive policy.* Outside of the consortium researchers and the governance committee, the greatest number of partnerships and collaborations formed were with national governments (81.8%), educational institutions (81.8%), non-governmental organizations (72.7%), research institutions (63.6%), scientific community (54.5%), regional government (54.5%), civil society (45.5%) and food industry (36.4%). Buy-in and support to carry out the program objectives was contingent on governmental support. For example, the Minister of Health in Costa Rica deemed the project as a public health priority which facilitated with data collection in grocery stores. The Minister of Health in Paraguay contributed additional human resources and support to carry out the research. Despite the strong partnerships formed, consortium researchers found the communication lines with the government were often unclear and required multiple points of contact in order to reach an individual of interest.

*Consortium researcher experiences with partnerships.* All consortium researchers reported a high level of satisfaction (very satisfied or satisfied) with their involvement in the consortium which led to engaged and activated partnerships throughout the program. All researchers felt respected, established trust for others and gained confidence to overcome challenges. One consortium researcher identified collaborators as friends. Furthermore, all researchers of the consortium understood the beliefs or values of each partner’s organization. The majority felt that communication with other researchers was consistent, transparent and open. Overall, 90.9% of researchers felt that common goals were shared and 81.8% felt that other researchers were committed to the research and consortium goals. Ultimately, the formation of a funded LAC consortium fostered a supportive and productive culture amongst collaborators and team researchers that led to high satisfaction levels, loyalty and commitment to advance sodium reduction research.

*Partnership challenges.* Misaligned expectations and team turnovers challenged partnerships. Select consortium researchers where disappointed when only one regional social marketing plan was created that required adaptations to specific countries when their expectation was that they would have the opportunity to generate country-specific social marketing plans. Most consortium countries had stable leadership over the grant period; however, Paraguay experienced changeover with research team members which resulted in delayed productivity due to recruitment and the duplication of training efforts. In contrast, another country experienced frequent government changes which resulted in shifts of political views and focus. In another situation, Brazil experienced logistical issues related to distribution of grant funds which limited Brazil’s participation in program objective 1B.

#### 3.1.3. Outcome 3: Equity, Diversity and Inclusivity

Principles of equity, diversity and inclusivity were incorporated into the funding program. All consortium countries had equal access to funds, resources and training to support the research. All consortium countries were from a LMIC and they were supported by researchers from high income countries. Researchers, public health officers, non-governmental representatives, scientists, academics and trainees had diverse skillsets, expertise and represented different geographical areas. The team was diverse in gender, capacity, career stage and had varying access to resources. Three out of 5 country leads identified as women and there was a high proportion of women who led research activities within the countries. In contrast, most government partners with a decision making role were men. This underscores a need to build capacity, confidence and opportunities for women to hold these senior positions. Consortium researchers were at various stages of their career, which allowed for mentorship, training and development of scientific and technical skillsets. Additionally, 25 trainees consisting of undergraduate (*n* = 7), graduate (*n* = 16) and postdoctoral fellows (*n* = 2) worked on the consortium research. The inclusion of trainees enabled the funding program to build capacity in the next generation of public health researchers.

Resources, training and development materials were translated into Spanish. Simultaneous interpretations into Spanish were made available for webinars, team meetings and virtual courses. At the onset of KT training, consortium researchers identified commonly used KT terms in North America, such as “audience”, “agents of change”, where the original meaning in English was different in Spanish. For comprehensive purposes, the KT training materials and workbook were revised to add definitions with illustrative examples on commonly used KT terms.

All consortium countries had equal access to funds, resources and training to support the research. There were also equal opportunities to disseminate research. However, in at least one instance there were disagreements on authorship when manuscripts were submitted for publication at the end of the funding period. Researchers were disappointed when they were excluded from publications. Having an a priori authorship agreement could have increased transparency and fairness when opportunities to publish arose.

#### 3.1.4. Outcome 4: Consortium Researchers Gained Confidence and Enhanced Capacity to Conduct Research to Address Public Health Nutrition Issues in LAC

*Quantitative and analytic skills.* One of the most significant achievements of the funded consortium was training opportunities that strengthened scientific capacity in the participating LAC. Scientific development opportunities related to quantitative data collection and analysis strengthened competencies and established new expertise that can be applied to support numerous public health nutrition priorities. The University of Toronto, Ontario Tech University and Laval University in Canada, University of South Florida in the United States, and the University of Liverpool in the United Kingdom, led training to strengthen knowledge and skills related to protocols, procedures, data collection and analysis techniques for research program objectives 1A, 1B, 2, and 3. These activities resulted in 90.9% of consortium researchers reporting enhanced skills and capacity to collect data and manage large data sets. Capacity development extended to leadership, networking, project management, communication and knowledge translation. All (100%) consortium researchers were satisfied or very satisfied with the training and development sessions offered in the research program. Table 1 outlines the training and skillset development observed during the grant.

*Social marketing and qualitative research.* Many consortium researchers found the social marketing training and development to be the most novel and educational. At the onset of the grant, researchers had varying levels of experience in social marketing, with a majority being introduced to the concept for the first time. Over 26 researchers enrolled in the initial social marketing training course with an 85.0% completion rate. The training had a focus on qualitative research and social marketing principles, led by researchers from the University of South Florida. Several consortium researchers indicated: “I learned the most in this [social marketing] objective”. Furthermore, consortium researchers expressed a deeper appreciation for the social marketing research experience beyond training and knowledge creation, which included the formation of meaningful collaborations and a sense of community. A partnership with the Association Latin Americana de Mercadeo Social, further supported the development of social marketing skills and stimulated collaborations with other LAC social marketing researchers.

*Knowledge translation skills.* The evaluation survey found that 90.9% of researchers reported a significant improvement in their knowledge and skills related to KT principles, concepts and their application [51]. For a majority of consortium researchers, KT was a new concept. Researchers reported that the KT workbooks helped them identify target audiences, generate strategic and impactful KT activities, and demonstrate that opportunities for information sharing can occur at multiple time points throughout the project cycle. The training led to practice changes for some. One researcher expressed “…when we have any research grants for small projects that would be funded … an idea [from the] IDRC [grant] is this communication plan … this knowledge transfer is something that is very important for presenting the results, making the results easier to understand for any audience”. Overall, 81.9% of researchers also agreed or strongly agreed that they enhanced their skills in preparing and submitting manuscripts to peer reviewed journals. At the conclusion of the funding period, consortium researchers generated numerous outputs to various target audiences, related to the consortium research activities, in the form of peer reviewed publications, meetings with decision makers and partners, technical reports, policy briefs and presentations (Table 2).

### 3.2. Intermediate Outcomes

The researchers’ dedication enabled them to achieve several intermediate outcomes, defined by the logic model (Figure 1), which were not expected during the grant period since they often take time to achieve. In Argentina, Costa Rica and Paraguay strong governmental support led to policy changes that informed their national plans on NCDs. In particular, the office of the Minister of Health in Peru added sodium reduction to their political agenda after communications with the consortium researchers, which was a notable achievement since in the past anemia and diabetes were the primary government priorities. In Peru the research was used to evaluate nutritional labelling so that updates on front-of-package label policies could be made [52]. At the regional level, data on the sodium content of foods led to a commitment from PAHO during the funding period to develop a set of revised targets, which was completed in 2021. This data also led to updated sodium targets in Argentina [42]. A renewed and strengthened partnership with the food industry was a key driver to support sodium reduction in the food supply. In June 2019, Costa Rica’s Ministry of Health and CACIA, Cámara Costarricense de la Industria Alimentaria (Costa Rican Chamber of the Food Industry) renewed their alliance and commitment to continue with sodium reduction efforts. This partnership inovovled joint action plans on setting national sodium goals, updates to sodium targets for packaged food products, and future plans for sodium reduction.

National commitments to support and fund the implementation of social marketing program were achieved. In Paraguay, municipal governments used the qualitative research to inform a social marketing plan focused on banning saltshakers in food service establishments. Costa Rica obtained funding from Resolve to Save Lives to adapt the regional social marketing plan to their national context. Finally, the resources, training and outputs by the consortium researchers were leveraged to address other nutrition topics of public health concern and stimulated partnerships and collaborations. A six-month grant extension from the IDRC enabled researchers to conduct analyses on other nutrients associated with non-communicable diseases. This included the analysis of already nutrient data collected with FLIP-LAC. Analyses related to nutrients (e.g., energy, sugar, saturated fat), the overall nutritional quality of packaged foods, and use of low sodium claims on the front of package were condcuted. Finally, the Organization for Economic Co-operation and Development invited the Principal Investigator to participate in a case study focused on best practices to reduce excess sodium consumption to mitigate CVD. This was a major achievement for future research in Costa Rica as the country did not have collaborations with civil society.

## 4. Discussion

This program evaluation demonstrated that a funded research consortium was an impactful way to build capacity and collaboration, and to generate evidence to inform population wide public health policies and programs for dietary sodium reduction. Specifically, this program evaluation demonstrated that all consortium level objectives were achieved for the duration of the program. With the IDRC’s investment in a funded research consortium, a significant amount of country and regionally specific evidence was generated from the five Latin American country participants. Policy change is rarely based on a single empirical research study [53]; however, in a short period of time the funded consortium translated their research data to impact national strategies and single policies within each country. To our knowledge, this is the first multi-country government funded research consortium that was assembled to address sodium reduction policies in the Latin American region. Other multi-national Latin American consortiums focused on obesity [54], cardiometabolic risk factors [55] and neurodegenerative conditions [56] using data sets from population-based studies. Consortium-based research projects are emerging as an effective way to address complex public health issues on NCDs [54,55,56]. In the current consortium, researchers engaged in a participatory approach to multi-method data collection and analysis with training on social marketing and knowledge translation, which is unique to this consortium. Capacity development in social marketing is particularly important since discretionary sodium is a primary source in many LAC, making population-level behavior changes essential to decreasing sodium consumption and preventing NCD-related morbidity and mortality.

Actions to reduce population-wide sodium intakes require multiple interventions that work synergistically, if countries are to achieve the WHO global target of 30% sodium reduction by 2025 [57]. At least 75 countries have developed a national strategy for dietary sodium reduction. Interventions identified under these national strategies commonly included one of the WHO “Best Buy” interventions, which have an average cost-effectiveness ratio of ≤$100 per disability-adjusted life years averted in LMICs. In 2019, a review found that 96 countries globally had a national sodium reduction program; however, only 6 LAC (Argentina, Canada, Chile, Colombia, Costa Rica and the United States) had implemented one to date [20]. In LAC, many countries have implemented at least one sodium policy, but not all have implemented one of the WHO “Best Buy” interventions [58]. Overall, 38% of countries have implemented food reformulation policies (voluntary or mandatory), 38% had policies for public settings, 15% had front-of-package labelling policies, and none had a national education campaign [20]. Despite the gradual introduction of dietary sodium reduction policies and programs on political agendas in LAC and globally, only a few countries have reported reduced mean sodium intakes and, to our knowledge, no LAC have met the WHO’s global sodium target [20]. These findings demonstrate the need for the implementation of evidence-informed, effective and progressive policies. Collaborative consortium-based research is a plausible approach to support complex policies for a public health challenge such as dietary sodium reduction; as seen with another consortiums focused on applied mental health systems research to produce health policies and services in low-income countries.

The funded consortium examined in this program evaluation allowed researchers and policy decision makers to form a community of practice with a shared focus on sodium reduction public health policies. This was a strength of the funded consortium as the community of practice that was created fostered learning interactions, shared knowledge, training and development efforts. It also created a rich environment to understand and make sense of new knowledge and training efforts, outcomes observed when communities of practices have strong and mutually respectful relationships. In other fields, a community of practice model has been utilized to promote practice changes in the clinical settings [59] and to improve policy and practice in cancer control [60]. The funded consortium encouraged scientific productivity and professional growth through access to scientific and technical experts in sodium reduction. Self-directed learning, consultation with experts, and between-country collaboration enabled opportunities for learning and mentorship—factors leading to the achievement of consortium goals. This finding is consistent with another Canadian community of practice that focused on translating evidence related to renal healthcare in vulnerable communities into clinical practice and policies [61]. To date, the consortium researchers continue to work together as a community of practice to advance knowledge, disseminate peer reviewed publications and best practices in other public health areas. The consortium model should be considered for new opportunities to address other public health areas, including other nutrition policy priorities.

The funded consortium integrated KT training and strategies for all the research program objectives, which is one factor leading to consortium success. Funding agencies can provide mechanisms to promote knowledge exchange through agency mandates on KT. One study found that 18 of 23 national and international funding agencies described some aspect of KT in their mandate [62]. It is well documented that policy development and KT in LAC are complex and slow [62]. Policy is further impacted by barriers to adoption such as limited resources, poor communication and dissemination methods and a lack of capacity to understand technical data [24,62,63]. This calls for resource allocation to support knowledge translation, better “packaging” of research results and for researchers and policy makers to develop technical capacity in understanding scientific data [24]. A larger issue are failures to use research in policy-making that contributed to $200 billion of wasted funds, which is detrimental in LMICs where resources are scarce. One study found that 18 of 23 national and international funding agencies described some aspect of KT in their mandate [62]. This funded consortium resulted in almost 500 KT outputs through systematic planning efforts, including many published manuscripts, that reached several sectors involved in policies and program development and implementation. The early policy advances the consortium observed are likely attributable to this large number of KT outputs. A limitation identified with the current consortium is that stronger integrated KT plans may have further enhanced policy and program impact, which would allow stakeholders and decision makers to have greater involvement in research planning, further increasing the usability of the data. Future consortium projects should allocate time and resources to build KT plans at the beginning of the funding period, using tools like the KT workbooks, to maximize impact of the findings at an earlier stage of the grant.

The use of a logic model framework was a strength of the program, which guided consortium actions and supported this evaluation. Only a few published studies have used a program evaluation framework, where most evaluations relied on observation designs [64]. Existing literature has criticized the logic model for its failure in capturing contextual factors and its utility in large community-based projects and initiatives [65,66]. Consortium researchers developed the logic model through an iterative feedback approach to ensure Latin American perspectives on contextual program factors was accounted for, an approach that has been widely utilized in other program evaluations [65,67]. By embedding contextual analysis and collaborations in the construction of the logic model, this grounded the program’s outcomes with IDRC’s program strategy and reinforced recommendations for best practices on how future funded programs would be implemented to a LA context [66].

## 5. Conclusions

This program evaluation illustrates the benefits of collaborative work in research—carried out by a consortium of LMIC countries in Latin America, with the support of international technical assistance—to facilitate the development and implementation of dietary sodium reduction policies, with an ultimate goal of reducing the burden of hypertension and cardiovascular disease. The dedicated efforts of the multidisciplinary team generated local and regionally scientific evidence for decision-making in sodium reduction policies and programs, innovations in food systems, using innovative technological and methodological approaches. The consortium is a model for other public health interventions that require new techniques for food policy analysis and consumer behavior, as well as the dissemination of key findings. The findings of this evaluation can be applied to the development of future consortium research partnerships and activities designed to address the burden of NCDs in the region.

## Figures and Tables

**Table 1:** Consortium researchers’ training and skills set development ^1^.

Program Objective	Organized Training	Scientific Skills Acquired
**Objective 1A and 1B**	2 online sessions (Obj 1A) 2 online sessions (Obj 1B) Additional one-on-one training, as needed	Enhanced abilities to collect, analyze and manage large datasets with the FLIP-LAC app and database Conduct quality assurance measures, statistical analysis, and reporting and presentation of data Knowledge front of package labelling Knowledge of how to prepare for and conduct chemical analysis procedures for sodium
**Objective 2**	3 online sessions 2 face-to-face workshops 1 online course Additional one-on-one training, as needed	Knowledge of the principles and concepts of social marketing research Knowledge and skills in identifying target behaviors and populations Qualitative research skills: creating a qualitative interview guide, conduct interviews and focus groups, coding transcripts Translating the formative research into a social marketing strategy Skills in creating a Creative Brief Creativity
**Objective 3**	2 week face-to-face training (1 country) 1 face-to-face workshop Additional one-on-one training, as needed	Knowledge and training of the principles, concepts and the application of health and economic modeling using the PRIME model and IMPACT model.

^1^ Consortium researchers reported organized training and scientific skills acquired.

**Table 2:** Summary of the reach of KT outputs across different target audiences.

Summary of KT Outputs ^2^							
Program Objective	Government	Health and Education	Food Industry	Civil Society	Scientists	International	TOTAL
**Objective 1A**	26	9	10	41	34	16	136
**Objective 1B**	11	10	15	5	20	13	74
**Objective 2**	11	9	0	10	36	4	70
**Objective 3**	2	1	0	0	6	5	14
**Objective 4**	6	14	4	43	5	4	76
**Objective 5**	4	35	2	41	19	19	120
**TOTAL**	60	78	31	140	120	61	

^2^ KT outputs include peer reviewed publications, meetings with decision makers and partners, technical reports, policy briefs and presentations. KT outputs summarized may overlap across different audience sectors.

## Data Availability

Not applicable.
